# FoPA: identifying perturbed signaling pathways in clinical conditions using formal methods

**DOI:** 10.1186/s12859-019-2635-6

**Published:** 2019-02-26

**Authors:** Fatemeh Mansoori, Maseud Rahgozar, Kaveh Kavousi

**Affiliations:** 1 Database Research Group, Control and Intelligent Processing Center of Excellence, School of Electrical and Computer Engineering, University of Tehran, Tehran, Iran; 2 Complex Biological Systems and Bioinformatics Lab (CBB), Bioinformatics department, University of Tehran, Tehran, Iran

**Keywords:** Pathway analysis, Formal methods, Enrichment analysis, Differentially gene expression

## Abstract

**Background:**

Accurate identification of perturbed signaling pathways based on differentially expressed genes between sample groups is one of the key factors in the understanding of diseases and druggable targets. Most pathway analysis methods prioritize impacted signaling pathways by incorporating pathway topology using simple graph-based models. Despite their relative success, these models are limited in describing all types of dependencies and interactions that exist in biological pathways.

**Results:**

In this work, we propose a new approach based on the formal modeling of signaling pathways. Signaling pathways are formally modeled, and then model checking tools are applied to find the likelihood of perturbation for each pathway in a given condition. By adopting formal methods, various complex interactions among biological parts are modeled, which can contribute to reducing the false-positive rate of the proposed approach. We have developed a tool named Formal model checking based pathway analysis (FoPA) based on this approach. FoPA is compared with three well-known pathway analysis methods: PADOG, CePa, and SPIA on the benchmark of 36 GEO datasets from various diseases by applying the target pathway technique. This validation technique eliminates the need for possibly biased human assessments of results. In the cases that, there is no apriori knowledge of all relevant pathways, simulated false inputs (permuted class labels and decoy pathways) are chosen as a set of negative controls to test the false positive rate of the methods. Finally, to further evaluate the efficiency of FoPA, it is applied to a list of autism-related genes.

**Conclusions:**

The results obtained by the target pathway technique demonstrate that FoPA is able to prioritize target pathways as well as PADOG but better than CePa and SPIA. Also, the false-positive rate of finding significant pathways using FoPA is lower than other compared methods. Also, FoPA can detect more consistent relevant pathways than other methods. The results of FoPA on autism-related genes highlight the role of “Renin-angiotensin system” pathway. This pathway has been supposed to have a pivotal role in some neurodegenerative diseases, while little attention has been paid to its impact on autism development so far.

**Electronic supplementary material:**

The online version of this article (10.1186/s12859-019-2635-6) contains supplementary material, which is available to authorized users.

## Background

Analysis of gene expression experiments comparing two groups of samples (e.g., normal and diseased), typically results in long lists of differentially expressed genes (DEGs). These long lists of genes are often hard to be interpreted by researchers. As a result, some methods have been developed to transform the gene expression data into meaningful sets. An example is to identify the set of genes that function in the same pathway which is commonly referred to as pathway analysis. This analysis is appealing to researchers for two reasons: first, grouping thousands of genes by the pathways in which they exist and involve, reduces the complexity to some hundred pathways; second, it facilitates identifying gene signaling networks relevant to a given condition which can help in understanding the mechanisms of diseases [1, 2], develop better drug production [3–5], personalize drug regimens [5, 6], etc.

The pathway analysis methods usually use two types of data as inputs: the experimental data, like gene expressions obtained when comparing two conditions and the pathway knowledge, which was previously known and stored in pathway databases. There exist several pathway databases providing collections of pathways for various organisms, most of which are drawn manually and updated regularly. Examples of these databases include KEGG [7], BioCarta/NCI-PID [8], PANTHER [9] and Reactome [10]. Pathway analysis tools use one or more pathway database(s) as their input and identify the pathways that are most relevant to a given condition. For more information regarding the pros and cons of various pathway analysis methods, please refer to the review published by Khatri et al. [11].

The pathway analysis methods are classified into two groups according to their strategy for incorporating the pathway data into their analysis: The first group considers pathways as simple gene lists [12–18] and the second group incorporates pathway topology in the analysis [19–26]. The former is usually referred to as ‘gene set based’ approach, and the latter is referred to as ‘Pathway Topology-based’(PT-based) approach. PT-based approach adds pathway topology in the analysis for utilizing the correlation between pathway components. The first proposed PT-based method was named Pathway-Express, as part of the Onto-tools suite [19]. Following that, some PT-based methods have been proposed. A comparison of PT-based methods is made by Mitrea et al. [27] with respect to their inputs, output, and analysis strategies.

Most PT-based methods [20, 24] model the biological pathways as simple graphs. They model genes as nodes and interactions among them as directed edges between nodes. This kind of modeling has some limitations: First, simple graphs are limited in describing all types of relations among genes involved in the same interaction. As some examples: (a) If a protein has some activators and inhibitors, an inhibitor may prevent the activation of the protein by each of its activators. Methods that use graphs to model signaling pathways use + 1 weight edges for activation interactions and − 1 weight edges for inhibition interactions, which does not accurately model the reality. (b) The condition where some genes together activate a gene; second, assume that a pathway is activated through a single receptor. If that particular receptor is not produced, the pathway will be probably completely shut off [20]. This problem is not addressed correctly by the graph modeling of signaling pathways; third, a simple graph is unable to model the concurrent and stochastic behavior of biological pathways.

Due to the similarity between biological systems and distributed systems studied in computer science, modeling techniques developed in formal methods can be applied to biological systems as well [28]. Formal methods are techniques for specification, verification, and analysis of systems. Systems are described rigorously by formal languages that help to reduce any ambiguity in the system specification. Once a model is constructed, it can be translated into a computer program for simulating the system under specification. This program can be used for reasoning and analyzing the system, predict the behavior of the system with some initial conditions, validating new experimental result, and identifying the inputs or parameters of the system enforcing a desired behavior [29]. Regev et.al. [30] were the first to propose considering signaling pathways as distributed computer systems. Since then, there has been a successful development in using formal methods in analyzing signaling pathways [31–33]. However, the objective of them is to model specific pathways to describe and analyses their dynamics rather than finding the most impacted signaling pathways in a given condition, the primary objective of this study. In this study, signaling pathways are formally modeled initially, and then model checking is used to find the likelihood of perturbation for each pathway in a given condition. FoPA tool is implemented based on this approach. Model checking is an automatic verification technique for finite state concurrent systems that helps to check whether a system model meets specified properties, by exhaustively exploring all possible executions of the system. In addition to the widespread application of this technique for ascertaining the correctness of distributed systems in computer science, it has recorded a remarkable success in analyzing biological signaling pathways [34, 35]. To the best of the authors’ knowledge, the proposed approach is the first attempt to use model checking to identify the pathways that are significantly affected in a given condition.

## Methods

In this section, first, some related basic concepts and primary definitions are briefly explained and then proposed approach is described in more details.

### Preliminaries

Probabilistic model checking is a variant of model checking used for analyzing systems that exhibit probabilistic behavior. A probabilistic model checker requires (a) a formal description of the system (formulated in some precise mathematical language), and (b) the specifications of one or more desired properties of that system in temporal logic (e.g., CTL or LTL). A model is typically a state-transition structure in which each state represents a configuration, and the transitions represent the evolution of the system from one configuration to another. In probabilistic model checking, the models are probabilistic (typically variant of Markov chains), in the sense that they are augmented with a probability of making a transition between states. As an endpoint, a probabilistic model checker returns “yes” or “no” indicating whether or not each property is satisfied, or the probability of some properties of the model, based on a systematic and exhaustive exploration of the model.

PRISM [36, 37] is a probabilistic model checker used for formal modeling and verification of quantitative properties of systems that exhibit random or probabilistic behavior. It can be used for analyzing different types of probabilistic models: continuous-time Markov chains (CTMC), discrete-time Markov chains (DTMC) and Markov decision processes (MDP). Models must be specified with the PRISM language, a simple language, based on the Reactive Modules formalism [38]. Properties to be verified against these models are expressed in probabilistic extensions of temporal logic.

A model described in the PRISM language consist of a set of modules that can interact with each other. The state of each module is being represented by the value of a set of finite-ranging variables. The global state of the whole model is determined by the local state of all modules. The behavior of each module is described by a set of commands of the form:

$$ \left[a\right] guard\to pro{b}_1: updat{e}_1+\dots + pro{b}_n: updat{e}_n; $$

The symbol *a* is an action label used for synchronization. If a transition does not have to synchronize with other transitions, then no action label needs to be provided for that. The *guard* is a predicate over all the variables in the model. When the *guard* is true, the model is updated according to the transitions and their probabilities described in the updates. The transitions are specified by giving the new values of the variables in the module, possibly as a function of other variables. The primed variable is used to represent the new values for the variables [39].

The *P* operator in the PRISM property specification language is used to reason about the probability of an event’s occurrence. For computing the actual probability that some behavior of a model is observed, PRISM allows the *P* operator to take the following form: *P* =? [*pathprop*]. *pathprop* is a formula that evaluates to either true or false for a single path in a model that describes the desired behavior [40].

### Model checking based approach

**Fig. 1:**
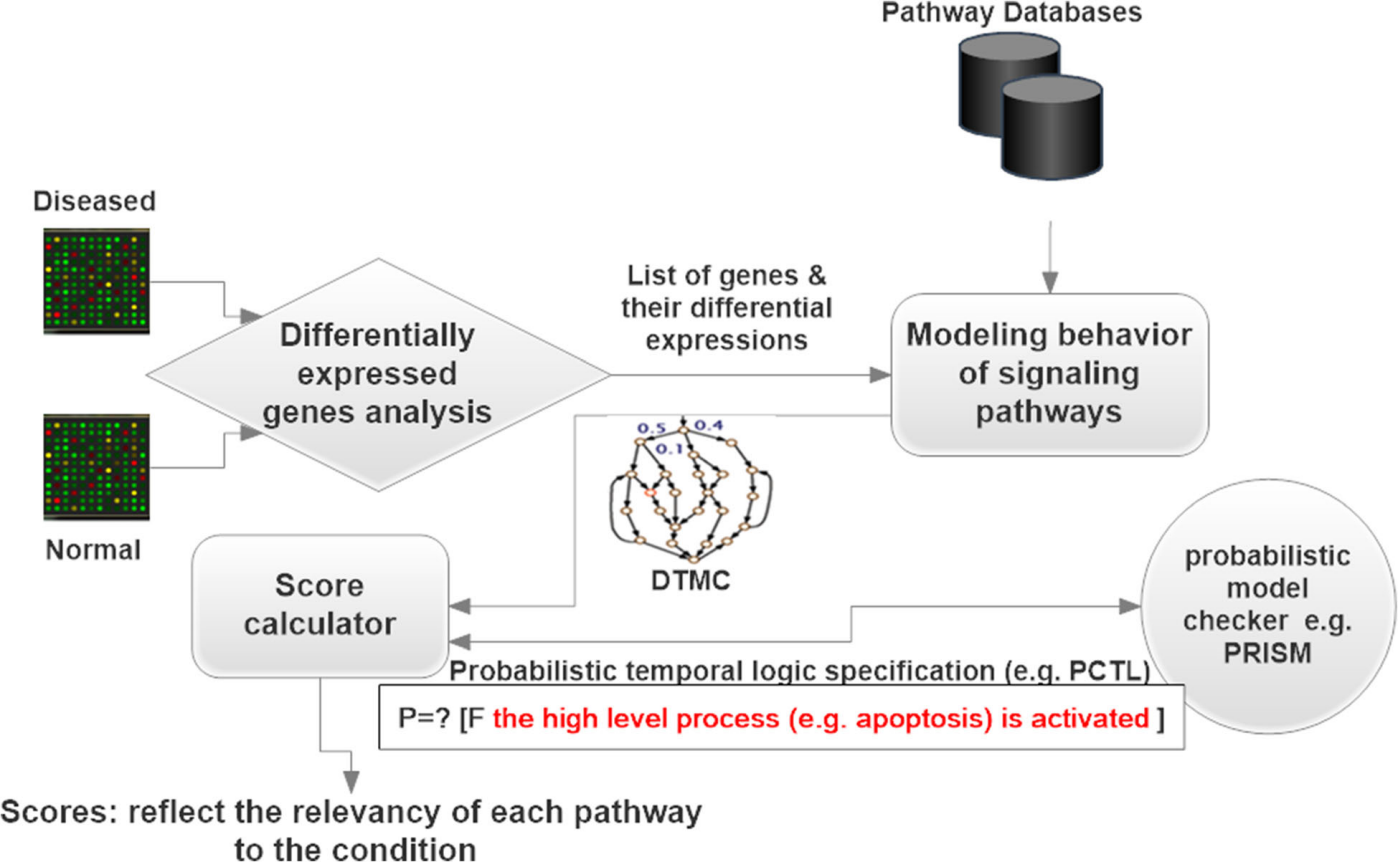
Architecture of the Model checking based approach: Model checking based approach requires a formal description of the behavior of the signaling pathways. The differential expression of genes between the conditions under study are used to estimate the parameters of the model or define the initial configuration. Once the model is specified by the proper formal language, it should be converted into discrete time or continues time Markova chains model which is usually done by the chosen model checking tool. After that, the model is given to score calculator which allocates a score to each pathway with the help of a model checking tool. For example, Score computation requests the model checking tool to compute the possibility of a cellular response activation

To understand the proposed approach, let’s formulate the query solved by the approach. Given that we have that two lists of genes R and R’ associated with the desired phenotype (i.e., normal and diseased) and a list of pathways (i.e.., all signaling pathways of KEGG) the query is to infer which one of the pathways are more related to the given phenotype. Figure 1 shows the proposed approach whose goal is to solve the query formulated above. The proposed approach requires a formal description of the behavior of the signaling pathways (formulated in some formal languages: i.e.., Petri net or PRISM modeling language). The differential expression of genes between the conditions under study are used to estimate the parameters of the model or define the initial configuration. Once the model is specified by the proper language, it should be converted into discrete time or continuous time Markova chain model which is usually done by the chosen model checking tool.

After that, the Markova chain model is given to score calculator which allocates a score to each pathway by executing its model with the help of a model checking tool. A Model checking tool receives a model of the system and checks whether this model satisfies given properties expressed in logical formulas. Therefore, in our application, the properties should be defined in a fashion that if they are satisfied with the model, the model could be considered related to the condition. An example of such properties is to check that whether a high-level process (e.g., apoptosis) in the given signaling pathway model is activated differentially when the model is initialized with the given differential expression of genes. The idea behind this property is that the signal transduction is a process that ultimately results in a cellular response.

The example property explained above is expressed by PRISM notation in Fig. 1, which means *the probability of the apoptosis response being active eventually in the future*. The probabilistic model checker is employed to check this property against the model. The probability returned by the probabilistic model checker is used to allocate a score to the pathway. The higher the score, the higher the relevancy of the pathway to the given phenotype (A toy example and more definitions and proofs are provided in Additional file 1).

### FoPA tool

**Fig. 2:**
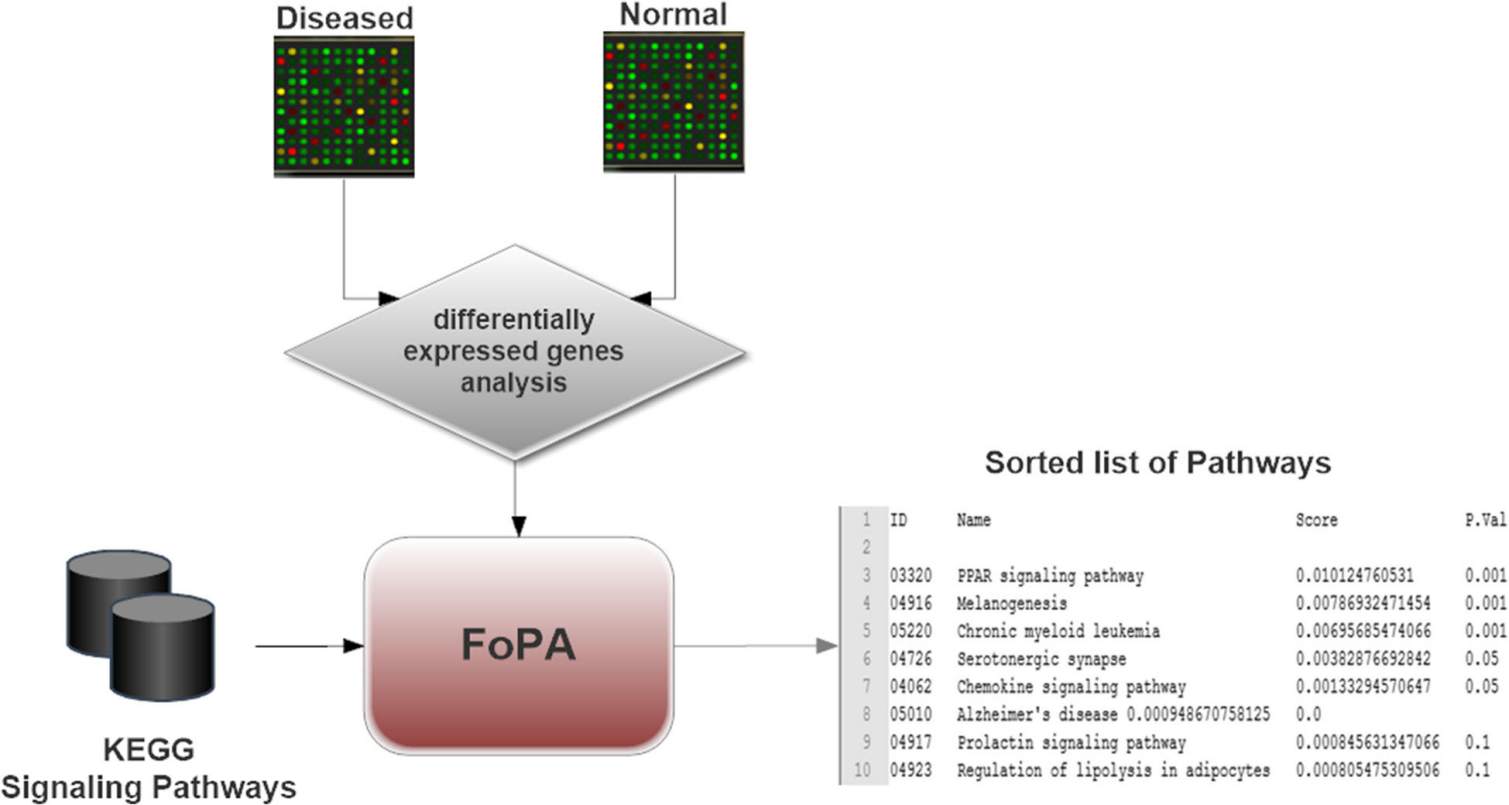
General overview of the FoPA: A list of differentially expressed genes and the signaling pathways of KEGG are fed to FoPA as inputs, and the output is a list of signaling pathways sorted according to their relevance to the differential genes

A pathway analysis tool named FoPA is introduced here, Fig. 2, using the approach proposed above. Gene expressions of the desired condition and its matched controls are converted to a list of differential gene expression, which is fed into FoPA as input along with the signaling pathways of KEGG. The output is a list of signaling pathways sorted according to their relevance to the desired condition.

**Fig. 3:**
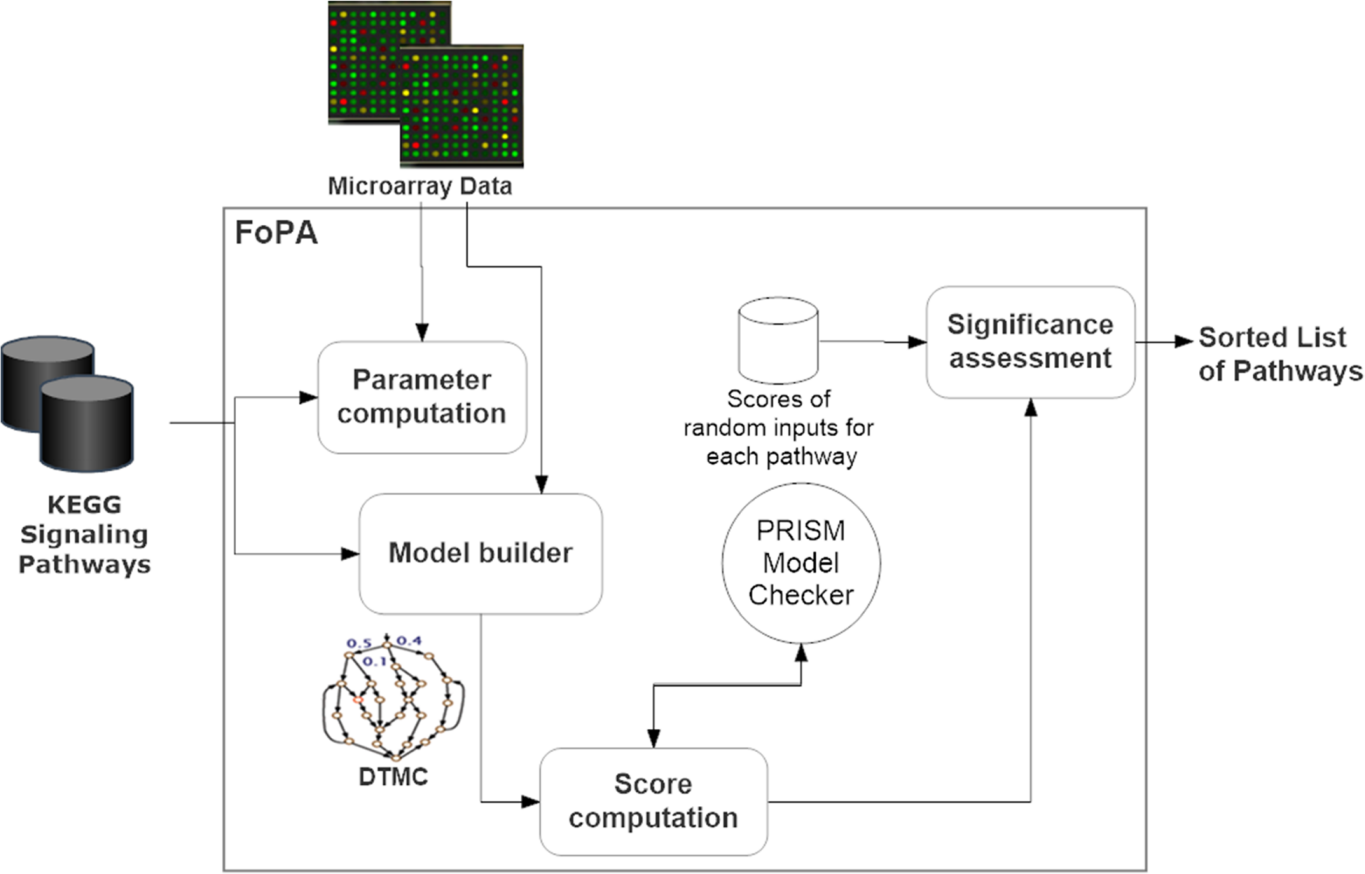
Different Parts of the FoPA: Model builder build a PRISM model for each pathway. The parameters of the model are estimated by Parameter computation using the KEGG pathways and gene expressions data. The Score computation defines the appropriate properties to check by PRISM. By computing the probability of these properties, a score is allocated to each pathway. An assessment of the significance of the measured score is done by Significance assessment

FoPA, Fig. 3, consists of four parts: Parameter computation, Model builder, Score computation, and Significance assessment. The Model builder constructs a PRISM model for each pathway. The model parameters (i.e., the probability of interactions and the initial state of the model) are estimated by Parameter computation using the KEGG pathways and gene expressions data. Score computation defines the appropriate properties to be checked by PRISM [37]. By computing the probability of these properties, a score is allocated to each pathway. This score is intended to reflect the relevancy of the pathway to the condition under study. However, this score can take place just by chance. Thus, an assessment of the significance of the measured score is required, which is done by Significance assessment.

### Model builder

KEGG signaling pathways consists of proteins/genes (throughout this paper, gene is used instead of protein and means protein-coding gene), small molecules and their interaction which includes but not limited to activation, inhibition, phosphorylation, dephosphorylation, and expression. They are represented by KGML format which is an XML representation of KEGG pathway maps. Model builder converts the KGML files into a formal model that can be executed by the PRISM tool.

Before building a PRISM model, some editing steps in KGML representation of signaling pathways have to be taken. First, in cases that several nodes are annotated with the same gene symbol, they are merged into a node, sharing all incoming and outgoing edges of the original nodes. Next, nodes representing small molecules and other non-gene parts are removed in a fashion that the parents and children of such a node stay connected.

For modeling the KEGG pathway by PRISM language, a variable is assigned to each gene. This variable indicates the state of the gene which can take on six values where three of them correspond to no expression, expression, and differentially expression of the gene relative to the control expression level (e.g., as measured in normal tissue) and encoded as 0, 1, and 2, respectively. The value − 1 for the variable representing a gene indicates that the variable is not initiated in the model yet. The values 3 and 4 belong to the activated states of the genes. When a gene is activated, it will move from one of its states 1 or 2 to the states 3 or 4. It will move to state 4 if it is differentially activated. A gene is differentially activated if it is a member of the differentially expressed genes and is activated, or it is activated by one of the members of differentially activated genes. Otherwise, it is not differentially activated and will move to state 3. By this type of modeling, the amount that a pathway is affected by the differentially expressed genes is considered. Interactions in KEGG pathways are modeled with PRISM commands where the details of them are tabulated in Table 1.

**Table 1:** PRISM commands modeled signaling pathway

No.			PRISM Language Specification
(1)	A → B	Activation	[ ]*A* > 2 & (*B* = 1 | *B* = 2) → *prob*_*active*_ : (*B*^′^ = (*A* = 3 & *B* = 1) ? 3 : 4) + (1 − *prob*_*active*_) : (*B*^′^ = 0);
(2)	A ⊣ B	Inhibition	[ ]*A* < 3 & (*B* = 1 | *B* = 2) → *prob*_*inhibit*1_ : (*B*^′^ = *B* + 2) + (1 − *prob*_*inhibit*1_) : (*B*^′^ = 0); [ ]*A* > 2 & (*B* > 2) → *prob*_*inhibit*2_ : (*B*^′^ = *B* − 2) + (1 − *prob*_*inhibit*2_) : *B*^′^ = 0;
(3)	A $\overset{+p}{\to }$ B	Phosphorylation activation	[ ]*A* > 2 & (*B* = 1 | *B* = 2) → *prob*_*active*_ : (*B*^′^ = (*A* = 3 & *B* = 1) ? 3 : 4) + (1 − *prob*_*active*_) : (*B*^′^ = 0);
(4)	A ⊣^+*p*^ B	Phosphorylation inhibition	[ ]*A* < 3 & (*B* = 1 | *B* = 2) → *prob*_*inhibit*1_ : (*B*^′^ = *B* + 2) + (1 − *prob*_*inhibit*1_) : (*B*^′^ = 0); [ ]*A* > 2 & (*B* > 2) → *prob*_*inhibit*2_ : (*B*^′^ = *B* − 2) + (1 − *prob*_*inhibit*2_) : *B*^′^ = 0;
(5)	A $\overset{-p}{\to }$ B	Dephosphorylation activation	[ ]*A* > 2 & (*B* = 1 | *B* = 2) → *prob*_*active*_ : (*B*^′^ = (*A* = 3 & *B* = 1) ? 3 : 4) + (1 − *prob*_*active*_) : (*B*^′^ = 0);
(6)	A ⊣^−*p*^ B	Dephosphorylation inhibition	[ ]*A* < 3 & (*B* = 1 | *B* = 2) → *prob*_*inhibit*1_ : (*B*^′^ = *B* + 2) + (1 − *prob*_*inhibit*1_) : (*B*^′^ = 0); [ ]*A* > 2 & (*B* > 2) → *prob*_*inhibit*2_ : (*B*^′^ = *B* − 2) + (1 − *prob*_*inhibit*2_) : *B*^′^ = 0;
(7)	A → B	Indirect effect	[ ]*A* > 2 & (*B* = 1 | *B* = 2) → *prob*_*active*_ : (*B*^′^ = (*A* = 3 & *B* = 1) ? 3 : 4) + (1 − *prob*_*active*_) : (*B*^′^ = 0);
(8)		*A* is not activated nor inhibited by other genes	[ ] (*A* = 1 | *A* = 2) → *prob*_*init*_ : (*A* = *A*^′^ + 2) + (1 − *prob*_*init*_) : (*A*^′^ = 0);
(9)	initializing the variables	*A* ∈ *expressed genes*	[ ] (*A* = − 1) → *prob*_1_ : (*A*^′^ = 1) + *prob*_2_ : (*A*^′^ = 2)
		A ∈ differentially expressed genes	[ ] (*A* = − 1) → *prob*_1_ : (*A*^′^ = 1) + *prob*_2_ : (*A*^′^ = 2)

Activation, Phosphorylation activation, Dephosphorylation activation, and Indirect effect are all different types of activation in which gene *A* activates the gene *B*. Thus, they model the same as the Activation relation. Likewise, the Inhibition, Phosphorylation inhibition, and Dephosphorylation inhibition are all different types of inhibition in which gene *A* prevents the activation of gene B and they model the same as the Inhibition relations. The *prob*s are the parameters for the commands that are replaced with the appropriate values when the model is constructed. *A* and *B* in PRISM command are variables indicating the states of the genes *A* and *B* respectively

In the following, modeling of interaction and inhibition interactions are described, where the rest of the interactions are modeled similarly.

In an activation interaction (*A → B*), an active gene *A* will activate gene *B*. The command for this interaction is expressed as command (1) in Table 1. In this command, *A* and *B* indicate the variables for modeling genes *A* and *B*. If *A* is active, (i.e., it is in states 3 or 4) and *B* is expressed either differentially (i.e., it is in state 2) or not (i.e., it is in state 1), then *B* will be active with the probability *prob*_*active*_, while with the probability *1-prob* be not active. The gene *B* moves to state 3 if neither *A* (the activator gene) nor *B* (The activated gene) belongs to the differentially expressed genes and it moves to state 4 if either *A* or *B* or both belong to differentially expressed genes.

The inhibition interaction (*A* ⊣ *B*) indicates that *A* inhibits the activation of gene *B*, that is, if *A* is active, *B* will not be activated. This interaction is modeled with commands (2) in Table 1. The first command indicates that if gene *B* is expressed and gene *A* is not activated then the gene *B* will be activated with probability *prob*_*inhibit*1_. If gene *B* belongs to differentially expressed genes, then it moves to state 4, otherwise moves to state 3. The second command indicates that if both genes *A* and *B* are active, *A* cause the inactivity of gene *B* with probability *prob*_*inhibit*2_.

In addition to commands for modeling each interaction, different commands are defined to initialize each variable. The measurement errors of data are considered in these commands. The command (9) in Table 1 shows these initializations wherein *A* represents a desired gene.

### Parameter computation

The model built by the Model builder is parametric. These parameters are *prob*_*active*_ (the probability of activation commands), *prob*_*inhibit*1_ and *prob*_*inhibit*2_ (the probability of inhibition commands), *prob*_*init*_ (the probability of activating a gene, which is not activated nor inhibited by other genes in the pathway), *prob*_1_ and *prob*_2_ (the probability of initializing the variables representing gens) which are estimated by Parameter computation.

To estimate the probability of commands (*A-B*) pieces of evidence are combined which are as follows:

1 $$ {prob}_{active}= diff\left(A,B\right)\ast P(A)\ast P\left(A\to B\right) $$

2 $$ {prob}_{inhibit1}=P\left(A\dashv B\right) $$

3 $$ {prob}_{inhibit2}= diff\left(A,B\right)\ast P(A)\ast P\left(A\dashv B\right) $$

The *diff(A,B)* term in the Eqs. (1–3) is formulated in Eq. (4). The reason behind the ratios in this equation is that two class of genes is defined in FoPA: DEG (differentially expressed gene) and notDEG (not differentially expressed gene). So, there are three types of relationships considering these classes: (a) notDEG – notDEG, (b) notDEG – DEG, and (c) DEG – DEG. It is expected that when there are more DEG-DEG relations in a pathway compared to another pathway, the first one is more relevant to the condition under study. For example, let’s suppose that two pathways have the same number of DEG genes with different numbers of DEG-DEG relations. In the absence of other factors, the pathway with more DEG-DEG relations seems to be more relevant to the condition under study. Hence, a higher weight is assigned to DEG-DEG relations than DEG-notDEG relations, and likewise, DEG-notDEG relations are assigned higher weight than notDEG-notDEG relations. The relative values 1, 2, and 3 are chosen to weight these relations.

In *diff(A,B)*, the value of α can be arbitrarily selected from the interval [0,$\frac{1}{3}$]. This interval has been chosen so that the value of *diff(A,B)* does not exceed 1. Since the pathway’s score are used to compare the pathways, selecting an arbitrary value for *α* will not affect the results, because all pathway’s score are changed by a factor of *α*. In FoPA, the value of α has been selected equal to $\frac{1}{6}.$

4 $$ diff\left(A,B\right)=\left\{\begin{array}{c}0<\alpha <\frac{1}{3},\kern0.5em A\kern0.5em an\mathrm{d}\ B\  are\  not\ differentially\ expressed\kern0.5em \\ {}2\alpha, \kern0.75em A\  or\ B\  is\ differentially\ expressed\kern2.75em \\ {}3\alpha, \kern0.75em A\  an d\ B\  are\kern0.5em differentially\ expressed\kern0.75em \end{array}\right. $$

By the second term in Eqs. (1, 3), *P(A)*, the amount of change of the gene *A* between two conditions of interest is of concern by using the *moderated t*-score [41] of this gene as follows:

5 $$ P(A)=\left|T(A). Fn(A)\right| $$

where *T(A)* is the *moderated t*-score of the gene *A* and the weight, *Fn*(*A*), is the function of the frequency of the gene *A* in the set of all pathways. The weight is defined such that reduced the contribution of the overlapping genes. The idea supporting this weighting is that whenever a differentially expressed gene appears in fewer pathways, it is assumed that particular gene reveals the evidence that those pathways are affected by the given condition. Therefore, the frequently appearing genes are assigned with a low weight close to the 0.0, while pathway-specific genes are assigned with a high weight close to the 1.0. Similar to what is done in Traca et al. method [16] the weight *Fn(A)* is defined as the normalized frequency of the gene *A* across all KEGG pathways in the scale of (0,1) as follows:

6 $$ Fn(A)=\sqrt{\frac{f(A)-\mathit{\min}(f)}{\mathit{\max}(f)-\mathit{\min}(f)}} $$

where *f(A)* is the frequency of gene *A*, *min(f)* is the minimum frequency of genes and *max(f)* is the maximum frequency of genes in the set of all pathways.

The third term in Eqs. (1) and(3), is the probability of interactions (*A → B*) and (*A* ⊣ *B*). To estimate these probabilities, the rational assumption is that the more the number of pathways in which *A* and *B* interact with each other than the number of pathways in which they have not interact with each other, the more the likely the interaction of *A* and *B*. Therefore, each pairwise interaction in the set of allpathways is checked against all the pathways in KEGG database. To estimate the probability of (*A → B*) interaction, the number of pathways, where there exist *A → B* are counted. The number of pathways where both *A* and *B* exist but have no activation association is between are also counted. Likewise, the activation interaction is replaced by inhibition to estimate the probability of inhibition interactions. Eqs. (7, 8) reveal how the probability of activations and inhibitions interactions are estimated, respectively.

7 $$ P\left(A\to B\right)=\frac{\left|\left\{ pathway\ \epsilon\ KEGG\ \right|A,B,\left(A\to B\right)\epsilon\ pathway\ \right\}\mid }{\left|\left\{ pathway s\ \epsilon\ KEGG\ \right|\ A,B\ \epsilon\ pathway\right\}\mid } $$

8 $$ P\left(A\dashv B\right)=\frac{\left|\left\{ pathway\ \epsilon\ KEGG\ \right|A,B,\left(A\dashv B\right)\epsilon\ pathway\ \right\}\mid }{\left|\left\{ pathway s\ \epsilon\ KEGG\ \right|\ A,B\ \epsilon\ pathway\right\}\mid } $$

The *prob*_*init*_ parameter which is used in modeling the activation of gene *A* that is not activated nor inhibited by other genes in the pathway (Command (8) in Table 1) is set equal to *P(A).*

To estimate the *prob1*, *prob2* parameters available in the Command (9) in Table 1, suppose, where the error for determining differentially expressed genes is α, according to which the equation set (9) is introduced as follows:

$$ g\in expressed\ genes\to {prob}_1=\left(1-\alpha \right),{prob}_2=\alpha $$

9 $$ g\in differentially\ expressed\ genes\to {prob}_1=\alpha, {prob}_2=\left(1-\alpha \right) $$

To estimate *α*, let’s suppose that, a cut off equal to *v* is chosen for FDR adjusted *p*-values for discovering the differentially expressed genes. It means that there is a *v*% chance that we make the wrong decision. In other words, the gene discovered as differentially expressed is not differentially expressed with the probability *v*%. Accordingly, *α* is chosen equal to the cut-off value *v*.

### Score computation

Score computation defines PRISM properties to check against the formal models of pathways. These properties are sought to find the possibility that the final effector genes (final genes that trigger cell responses) are differentially activated; that is, they are in state 4. This property is defined in PRISM language as:

10 $$ P=?\left[F\ \left(g=4\right)\right] $$

where *F* is a temporal operator means eventually in the future, and *g* is a final effector gene. These probabilities are computed for every final effector genes of the pathway *path,* and their sum is used to assign a score to each pathway as follows:

11 $$ score={\sum}_{g\ \mathrm{is}\ \mathrm{a}\ \mathrm{final}\ \mathrm{effector}\ \mathrm{gene}\ \mathrm{of}\  path}P=?\left[F\ \left(g=4\right)\right]. $$

### Significance assessment

Pathway scores are intended to provide the amount of change incurred by the pathway between two phenotypes (e.g., normal and diseased). However, the amount of change can take place by chance. Consequently, an assessment of the significance of the measured changes is required.

To obtain the significance of the measured change the null distribution must first be estimated. The null hypothesis here is that the differential expression of the genes does not associate with the condition under study. Consequently, for constructing null distribution, the pathway scores for the situations where the random number of DE genes are scattered randomly in the pathway are of interest.

Thus, the null distribution is constructed by permuting the label of the normal and disease samples. This procedure generates samples under the assumption that no particular association between the gene differential expressions and phenotype exist. Class label permutations allowed to maintain gene-gene relations but remove the association between differential expressions of genes and the condition under study.

Thus to assess the significance of a pathway score, the sample labels are swapped *N*_*perm*_ times and the score is recalculated for these new samples. Finally, the significant of pathway score is obtained as follows:

12 $$ {P}_F=\frac{\sum_{perm}\mathrm{I}\left({Score}_{perm}\ge {Score}_{real\_ sample}\ \right)}{N_{perm}} $$

where I(.) is an indicator function, *path* _ *score*_*perm*_is the score of the pathway for each permutation, *path* _ *score*_*real* _ *sample*_ is the score of the pathway for the main data and *N*_*perm*_ is the number of permutations.

### Data analysis

All 36 datasets in the mentioned benchmark of the target pathway technique are available from the Gene Expression Omnibus (GEO) (details for each dataset are given in Additional file 2). These datasets are collected and normalized as ‘KEGGdzPathwaysGEO’ [42] and ‘KEGGandMetacoreDzPathwaysGEO’ [43] R packages. For all, a *moderated t-test* between disease and normal groups is performed by using the R limma package [44], followed by selecting genes with *FDR adjacent p-values* [45] less than *0.05* as differential.

## Evaluation

### Comparing FoPA with other existing methods

Assessing the correctness of any pathway analysis method in real experiments is a challenging task because a real gold standard has been not proposed yet. Lack of a definitive answer concerning the involvement of a given pathway in a given condition makes it impossible to calculate exact values for sensitivity, specificity, ROCs. Under such circumstances, it is best to compare the results of the desired pathway analysis method with other available and well-known methods.

Among available TP-based methods, the ones with available R scripts or packages for downloading are of concern in this study. These methods are compared by Bayerlova et al. [46]. In this comparison, centrality based pathway analysis (CePa-GSA) [24] indicates better results, therefore, FoPA is compared with it. Moreover, signaling pathway impact analysis (SPIA) [20] is chosen for comparison, because, it is the first introduced PT-based method and almost, all methods compare their results with it. Furthermore, some gene set based methods are compared by Tarca et al. [47] indicating that the pathway level analysis of gene expression (Plage) [14], Globaltest [12] and pathway analysis with down-weighting overlapping genes (PADOG) [16] outperform their counterparts. Because PADOG is newer and ranks the target pathways better than the other two, it is chosen for comparison here. In the following, these methods are described briefly.

CePa_GSA incorporates network centralities to weight gene-level statistics, and then these statistics transforms into pathway level statistics. For gene level statistics the absolute value of t-statistic is used as default, and the default function for computing pathway level statistics is *mean* function.

SPIA is combining two scores. For computing the first one, it is assumed the same as the simple ORA methods, that the number of DEGs in a given pathway follows the hypergeometric distribution. To obtain the second, so-called perturbation score, the pathway topology information is incorporated into the analysis. First, to each gene in a pathway a perturbation factor is assigned which is the logarithm of the fold-change (logFC) of this gene and the sum of perturbation factors of its direct upstream genes normalized by the number of all its downstream genes. The terms of the sum are weighted by the type of interaction between genes: 1 for activation and − 1 for inhibition. Next, the accumulated perturbation of each gene is computed by the difference between the perturbation factor of that gene and its observed logFC. Finally, the accumulated perturbations of the pathway’s genes are aggregated in total pathway accumulated perturbation. The two scores are then combined into a global score by using Fisher’s product test.

Gene set scores in PADOG are computed by first calculating moderated t-scores for all the genes, and then integrating the weighted moderated t-scores in a global gene set score. The weight of a gene in a gene set is down-weighted if it is involved in multiple gene sets. Thus, the higher weights are given to gene set specific genes, prioritizing their effect in the scoring.

### Target pathway technique

Most of the pathway analysis methods usually select a few real datasets for comparing their methods with others and then interpret the results either with the help of a life scientist or by searching the published literature. But since a large number of pathways are implicated directly or indirectly in any biological condition, authors may select specific literature as supporting evidence. Thus, this type of validation may lead to biased results.

A better assessment approach must eliminate human bias and be performed on a large number of datasets and conditions. The validation approach introduced by Tarca et al. [47] is reproducible, based on multiple datasets, and does not require an expert human evaluation of the results.

In this approach, multiple microarray datasets are used as a benchmark. Every dataset in this benchmark represents a particular disease coming from different tissues and laboratories. Each dataset has been linked to a defined pathway from the KEGG database which is considered to be the target pathway for that dataset. For example, a dataset comparing normal and cancerous colon would have ‘colorectal cancer’ as its target pathway. It is expected from any pathway analysis method to identify the colorectal pathway as affected and rank it close to the top. Methods are compared based on their performance in ranking the target pathways.

Based on the above explanations, the sensitivity of a method is defined as the median *p*-value of the target pathways over benchmark datasets (a lower p-value indicates higher sensitivity). The prioritization of a method is the medians of the rank percentage of the target pathways over benchmark datasets.

### False positive rate

The disadvantage of the target pathway technique is focusing on only one pathway for each dataset, whereas the behavior of a biological system may be governed by more than one pathway in a given condition. Because in reality, there is no a priori knowledge of all relevant pathways, the simulated false inputs (permuted class labels and decoy pathways) are chosen as a set of negative controls.

In the first, 50 trials are used wherein the class labels (e.g., normal, disease) of the actual samples are randomly permuted before the analysis. The percentage mean of the significant pathway subject to this null hypothesis is expressed as the false positive rate of the method. By using this null hypothesis, the expression levels are dissociated from the studied phenotypes while the gene-gene correlations are preserved. In the second technique, the simulated decoy pathways are chosen as a set of negative controls. Decoy pathways are generated using KEGG pathways. A decoy pathway maintains the structure of the KEGG pathway that is made from, with the difference that its genes are substituted with random genes from the set of all genes. Compared methods are run on both KEGG real and decoy pathways to check their ability to distinguish decoy from real pathways. The ROC curves are created by plotting the true-positive rate (the rate of the real pathways) against the false-positive rate (the rate of the decoy pathways) at various threshold levels. The area under these curves is defined as a measure of how methods can well distinguish between the decoy and real pathways.

### Consistent results for related datasets

Dong et al. [17] assumed that a successful method should produce consistent results for independent datasets under similar studying conditions. They select three independent datasets and performed enrichment analysis for them and then counted the number of overlapping gene sets that are significant in at least two datasets at a given rank threshold. Here, the same strategy is followed to test the performance of the FoPA under similar conditions. The analysis is performed for five independent colorectal cancer datasets (GSE4107, GSE8671, GSE9348, GSE23878, GSE4183). At a given rank threshold (e.g., top 10, 20, …, 50 significant pathways) and for each pair of datasets, the number of overlapping pathways that are identified relevant is counted. This value demonstrates the consistency between the results of the method for these five datasets.

Because of tumor heterogeneity nature of cancers including colorectal cancer, another test for consistency analysis is performed. In this test, instead of choosing independence datasets, a dataset with *N* sample is selected. This dataset is randomly resampled to obtain datasets of size n < N. The analysis is then performed on these new resampled datasets. Since these datasets are chosen from the same experiment, mehods should produce consistent results on them.

### Application of FoPA to real data samples

As an application of FoPA, it is applied to two real data samples. The first one is the colorectal cancer dataset used in the target pathway technique (GSE8671) and the second one is a list of autism-related genes identified by Rubies et al. [48].

### Systematic analysis of the possible bias

The *p*-values produced for each pathway by a pathway analysis method must be uniformly distributed in the interval [0,1] when the null hypothesis is true [49]. If the *p*-values are not uniformly distributed, the result of the pathway analysis method may be biased. For example, pathways that have p-values biased towards zero may often be falsely identified as significant.

In [49] an approach for constructing an empirical null distribution for analysing the systematic bias of the methods is proposed. In this approach, the expression data related to the control samples of some independent Alzheimer’s disease experiments are used. Half of these samples are randomly labeled as disease, and the rest are labeled as normal. This procedure is repeated many times to generate different groups of control and disease samples. Groups with fewer or more disease samples (e.g., 10 diseases and 20 normal or 20 diseases and 10 normal) are also generated to eliminate the effect of sample size. Then, the p-values of the KEGG signaling pathways are calculated for each group. These p-values should be uniformly distributed.

### The impact of pathway’s incompleteness and noise on FoPA’s performance

Currently, available pathway databases are not complete and may be noisy. To inspect how incompleteness and noise affect FoPA’s performance, they are mimicked by randomly removing or rewiring a portion (e.g., 10, 20, 30,40, and 50%) of interactions in a pathway.

By interaction removal, an interaction between genes is removed and by interaction rewiring the endpoint of an interaction is set uniformly to a new gene from the pathway. The edge removal and edge rewriting will not remove genes from the pathways but will make changes in the gene-gene interactions.

At each portion of edge removal or rewriting for each pathway, the procedure is repeated 100 times. The similarity measure for each pathway is computed as the number of generated pathways identified related or unrelated as the main pathway divided by the number of genrated pathways.

## Results and discussion

### Target pathway technique

**Fig. 4:**
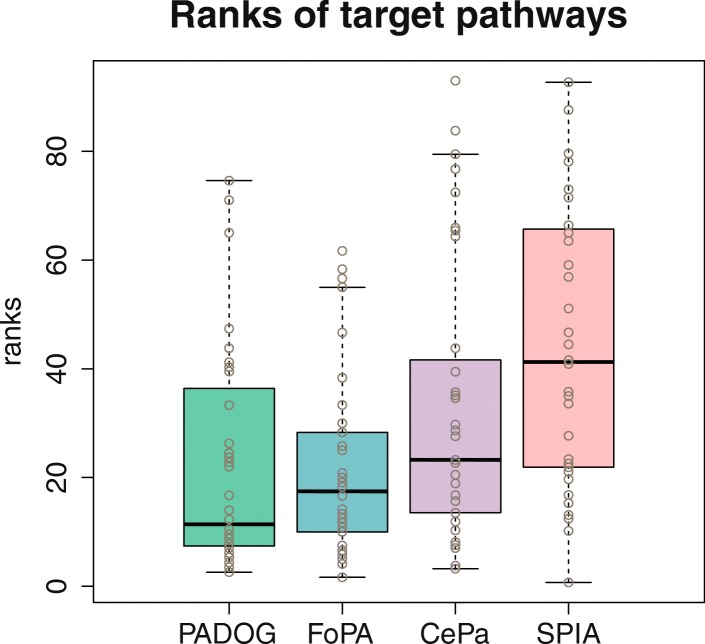
Comparing ranks of target pathways: Each box contains 36 data points representing the rank (%) of the target pathway in each method when using as input an independent dataset. Methods are ranked from best to worst according to the median rank

**Fig. 5:**
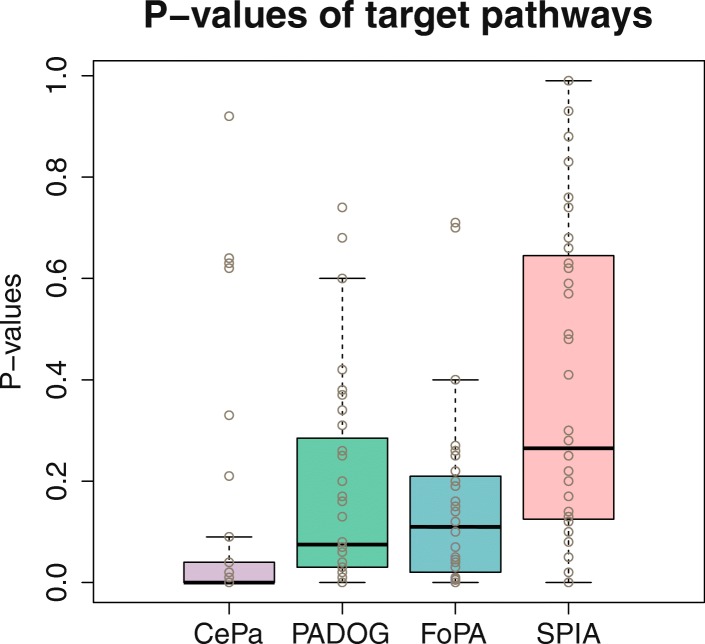
Comparing *p*-values of target pathways: Each box contains 36 data points representing the p-values of the target pathway in each method when using as input an independent dataset. Methods are ranked from best to worst according to the median p-value

The median of *p*-values and the median of the ranks of the target pathways over benchmark datasets are defines as sensitivity and prioritization of the methods. The results considering both the rankings and *P*-values of the target pathways associated with each dataset, Figs. 4 and 5, are assessed (for additional details see Additional file 3).

The summary of results for the four different methods based on the panel of 36 datasets is tabulated in Table 2. Out of the four compared methods, CePa ranks the 1st regarding sensitivity, while it ranks the 3rd regarding prioritization; PADOG ranks the 2nd regarding sensitivity, and the 1st regarding prioritization and FoPA ranks the 3rd regarding sensitivity and the 2nd regarding prioritization.

**Table 2:** Comparison between 4 methods regarding prioritization, sensitivity and the reciprocal ranks of the target pathways

	FoPA	PADOG	CePa	SPIA
mean reciprocal rank	**0.09**	**0.1**	0.06	0.07
rank median	17.5	**11.4**	25.25	41.25
rank mean	21.52	**21.91**	33.55	44.07
p median	0.11	0.08	**0**	0.27
p mean	0.15	0.18	**0.11**	0.38
Wilcoxon *rank*	Reference	0.9	0.008	5e-05
Wilcoxon *p*	Reference	0.45	0.02	0.0001

The table shows the statistics computed from the p-values and ranks of the 36 target pathways identified by each method. The mean reciprocal rank is the average of the reciprocal ranks of the target pathways computed by the equation $\frac{1}{N}{\sum}_{i=1}^N\frac{1}{{\mathit{\operatorname{rank}}}_i}$. The results of comparing the ranks of each method against FoPA (chosen as reference), using a paired Wilcoxon test are also included. The best value for each measure is shown in bold

The mean and mean reciprocal ranks of the target pathways are almost equal in PADOG, and FoPA suggested that on average FoPA ranks the target pathways as well as PADOG ranks them. This Wilcoxon signed rank test which is done on the rank and p-value of PADOG and FoPA also confirms this outcome and shows that there is no statistically significant difference between the distribution of ranks and *p*-values of FoPA and PADOG. Thus, it can be concluded that PADOG and FOPA are able to prioritize target pathways with high sensitivity when compared with CePa and SPIA.

### False positive rate

Two experiments are used to measure the false positive rate of the methods. In the first, 50 trials of the original datasets with randomly permutated class labels (e.g., normal, disease) are used. The percentage mean of the significant pathway subject to these random datasets is considered as the false positive rate of the methods. In the second, the simulated decoy pathways generated by connecting random genes using the same network structure as the KEGG pathways, are chosen as a set of negative controls. Compared methods are run on both KEGG real and decoy pathways to check their ability to distinguish decoy from real pathways.

**Fig. 6:**
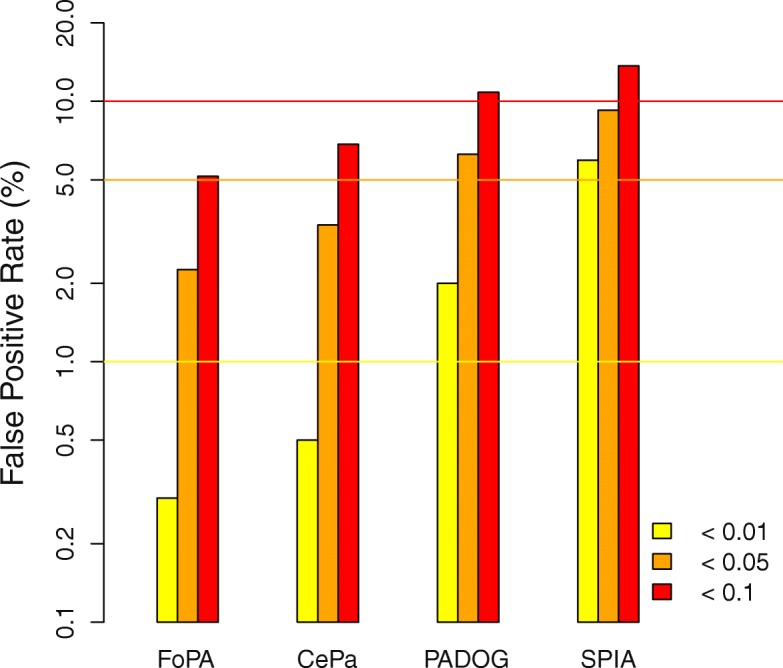
Comparing false-positive rates produced by four methods: The percentage of all pathways found significant at different significance thresholds is reported for each method with a vertical bar (the scale is logarithmic). The horizontal lines indicate the expected number of false positives at each threshold. Methods are ranked from best to worst according to their false positive rates

The false-positive rate of all methods for different significance threshold produced by analyzing 50 permuted versions of original datasets is shown in Fig. 6. The percentage of all pathways found significant at different significance thresholds is reported for each method with a vertical bar which indicates that the FoPA false-positive rate is less than that of other methods.

**Fig. 7:**
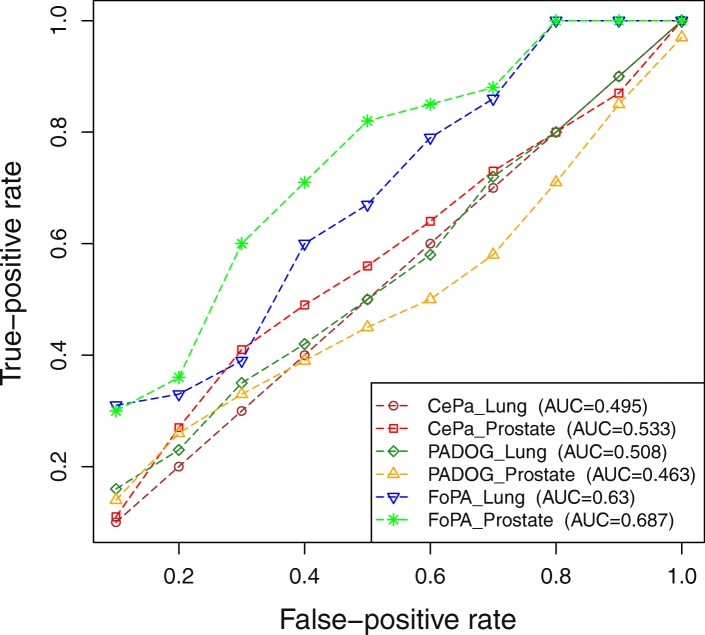
Distinguishing real and decoy pathways with FoPA, PADOG and CePa: Each line shows the receiver operator characteristic for distinguishing real and decoy pathways. Two datasets prostate cancer (GSE6956C) and Non-small cell lung cancer (GSE18842) are fed into the methods as desired conditions

The results of three methods FoPA, CePa and PADOG in ranking decoy and real pathways are shown as ROC curves in Fig. 7. Two datasets (GSE6956C, GSE18842) are chosen out of 36 datasets to feed into the methods as inputs. No matter which datasets are selected, the decoy pathways should not appear in high ranks of the results for the input dataset. The results here have illustrated that the area under the curves ROC in FoPA is greater for both datasets. That is FoPA outperforms PADOG and CePa in distinguishing real from decoy pathways.

### Consistency result for independent experiments

**Fig. 8:**
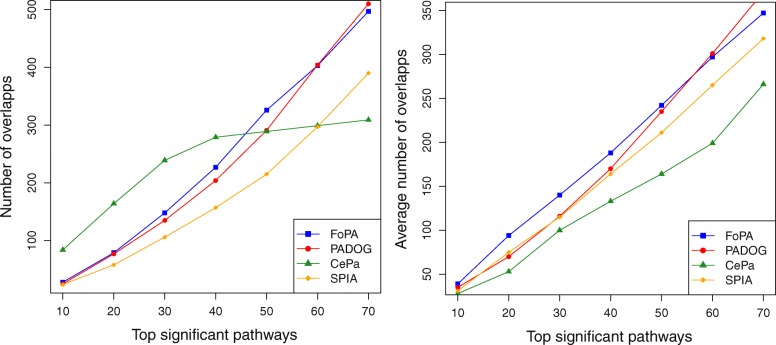
Overlaps among relevant pathways identified by different methods at the different rank threshold: **a** Scatter plots of the number of overlapped pathways identified in five colorectal cancer datasets. **b** Scatter plot of the number of overlapped pathways identified in resampled datasets of a colorectal cancer dataset (GSE8671) at the different rank threshold

An analysis is performed for five independent colorectal cancer datasets (GSE4107, GSE8671, GSE9348, GSE23878, GSE4183). The results of methods for these datasets are assessed at multiple rank thresholds. At a given rank threshold (e.g., top 10, 20, …, 60 significant pathways) the overlapping pathways between the results of each pair of datasets are counted which is shown in Fig. 8a. In this experiment, FoPA identifies more overlaps than PADOG and SPIA at almost all rank thresholds. CePa recognizes more overlaps in ranks 10, 20, 30, 40. However, it seems the recognized list by CePa as shown in Table 3, contains false-positive (The complete results can be found in Additional files 4 and 5).

**Table 3:** Overlapped pathways in the top 10 significant pathways found by each method in five colorectal cancer datasets (GSE4107, GSE8671, GSE9348, GSE23878, GSE4183)

	Overlapping Pathways in four colorectal cancer datasets		Overlapping Pathways in three colorectal cancer datasets	
	KEGG ID	Name	KEGG ID	Name
FoPA	04962	Vasopressin-regulated water reabsorption	04976	Bile secretion
	04960	Aldosterone-regulated sodium reabsorption	04064	NF-kappa B signaling pathway
			04978	Mineral absorption
			05010	Alzheimer’s disease
PADOG	05210	Colorectal cancer	05211	Renal cell carcinoma
	05222	Small cell lung cancer		
CePa	04310	Wnt signaling pathway	00051	Fructose and mannose metabolism
	04514	Cell adhesion molecules (CAMs)	00563	Glycosylphosphatidylinositol (GPI)-anchor biosynthesis
	00430	Taurine and hypotaurine metabolism		
	04810	Regulation of actin cytoskeleton		
	04722	Neurotrophin signaling pathway		
	00250	Alanine, aspartate and glutamate metabolism		
	00983	Drug metabolism - other enzymes		
	05414	Dilated cardiomyopathy (DCM)		
SPIA	4512	ECM-receptor interaction	3320	PPAR signaling pathway
			4062	Chemokine signaling pathway
			4110	Cell cycle
			4725	Cholinergic synaps

In Table 3, the *‘colorectal cancer*’ and *‘Renal cell carcinoma*’ are overlapped respectively among the significant pathways found by PADOG in four and three of the colorectal cancer datasets. However, the null distribution analysis performed in [49] has shown that *p*-values produced by PADOG for the *‘colorectal cancer*’ and the *‘Renal cell carcinoma*’ pathways are biased towards zero, that makes these pathways detected as significant regardless of input dataset.

FoPA found the *‘Vasopressin-regulated water reabsorption*’ and the *‘Aldosterone-regulated sodium reabsorption*’ pathways overlapped between the significant pathways in four colorectal datasets.

The presence of vasopressin receptors was reported in transformed epithelial cells, as well as in a wide panel of human tumor cell lines [50]. In addition, the expression of vasopressin receptor is confirmed in commercially available colon tumor samples [51]. Moreover, desmopressin, a synthetic analogue of vasopressin, has shown significant antitumor activity in preclinical murine models of colorectal cancer [52]. Therefore, finding the *‘Vasopressin-regulated water reabsorption*’ pathway as an overlapped pathway in colorectal cancer datasets is in line with other findings.

We refer to the research done by Guo et al. [53] to indicate that the second overlapped pathway, *‘Aldosterone-regulated sodium reabsorption*’, is also consistent with the other findings. In this research, some colorectal datasets are integrated to elucidate the potential key candidate genes and pathways in CRC. The *‘Aldosterone-regulated sodium reabsorption pathway*’ is among the candidate pathways that have been identified as common in CRC.

### Consistency results for resampling datasets of one experiment

From the five colorectal cancer dataset above, GSE8671 is chosen, since it has more samples than the others. This dataset is randomly resampled to obtain sub-datasets of size 8, 16, and 32. This procedure is repeated 50 times to create different groups of samples. The result of the four methods for these datasets is assessed for consistency of results. At each given rank threshold the average number of overlapped pathways are shown in Fig. 8b. As it clear, the average number of overlapped pathways identified by FoPA is more than that of other methods in almost all rank thresholds.

### Pathways ranking on colorectal cancer dataset

As an example, the results of FoPA are analyzed on one of the datasets used in the target pathway technique and shown it is consistent with other available studies. This dataset compares 32 pairs of samples collected from colorectal adenomas with those of normal mucosa from the same individuals [54] using Affymetrix HG-U133 Plus 2.0. Microarray platform. This dataset is available via Gene Expression Omnibus (ID = GSE8671) (For details about the differentially expressed genes in this dataset, refer to Additional file 6). The *KIA1199* gene is reported as the most overexpressed gene in this study. There is an increasing body of evidence that suggests the involvement of this gene in cancer progression, metastasis and poor prognosis of patients with colorectal cancer [55]. Cancer data analysis indicates that the expression of *KIAA1199* and ‘*Wnt-signaling pathway*’ genes are correlated [56]. Thus, ‘*Wnt signaling pathway’* is likely to be relevant to the condition under study in this dataset. Four pathway analysis methods are compared regarding their ability to identify the ‘*Wnt signaling pathway’* as relevant to the present dataset. The results are tabulated in Table 4 (For complete results refer to Additional file 5). As illustrated, FoPA identifies the ‘*Wnt signaling pathway* as significant’ (*p*-value < 0.05) and with a lower rank than the other compared methods.

**Table 4:** The top 15 pathway retrieved by FoPA, PADOG, CePa and SPIA for the colorectal cancer (GSE8671) dataset

FoPA				PADOG			
Rank	KEGG pathway	KEGG ID	P_FoPA_	Rank	KEGG pathway	KEGG ID	P_PADOG_
1	Non-small cell lung cancer	05223	0.0064	1	RNA transport	03013	0.0001
2	Intestinal immune network for IgA production	04672	0.0194	2	Cell cycle	04110	0.0001
3	Aldosterone-regulated sodium reabsorption	04960	0.0194	3	p53 signaling pathway	04115	0.0001
4	Tight junction	04530	0.038	4	Progesterone-mediated oocyte maturation	04914	0.0001
5	p53 signaling pathway	04115	0.048	**5**	**Colorectal cancer**	**05210**	**0.0001**
**6**	**Wnt signaling pathway**	**04310**	**0.05**	6	Non-small cell lung cancer	05223	0.0001
7	Mineral absorption	04978	0.0679	7	Endometrial cancer	05213	0.01
8	Renin-angiotensin system	04614	0.067	8	Small cell lung cancer	05222	0.0299
9	Alzheimer’s disease	05010	0.07	9	Glioma	05214	0.0299
10	Vasopressin-regulated water reabsorption	04962	0.07	10	RNA degradation	03018	0.040
11	NF-kappa B signaling pathway	04064	0.07	11	Apoptosis	04210	0.040
12	Neurotrophin signaling pathway	04722	0.08	12	Prostate cancer	05215	0.040
13	Melanoma	05218	0.09	13	Prion diseases	05020	0.040
**14**	**Colorectal cancer**	**05210**	**0.1**	14	Gap junction	04540	0.050
15	Leukocyte transendothelial migration	04670	0.12	15	Pancreatic cancer	05212	0.050
				…	…		
				**28**	**Wnt signaling pathway**	**04310**	**0.2**
CePa				SPIA			
Rank	KEGG pathway	KEGG ID	P_CePa_	Rank	KEGG pathway	KEGG ID	P_SPIA_
1	Cell adhesion molecules (CAMs)	04514	0.009	1	Chemokine signaling pathway	04062	0.001
2	Taurine and hypotaurine metabolism	00430	0.009	2	Cell cycle	04110	0.001
3	Glycosylphosphatidylinositol (GPI)-anchor biosynthesis	00563	0.009	3	p53 signaling pathway	04115	0.001
4	Regulation of actin cytoskeleton	04810	0.009	4	ECM-receptor interaction	04512	0.001
5	Circadian rhythm	04710	0.009	5	Gap junction	04540	0.001
6	Neurotrophin signaling pathway	04722	0.009	6	Natural killer cell mediated cytotoxicity	04650	0.001
7	Alanine, aspartate and glutamate metabolism	00250	0.009	7	Fc gamma R-mediated phagocytosis	04666	0.001
8	Drug metabolism - other enzymes	00983	0.009	8	Cholinergic synapse	04725	0.001
9	Dilated cardiomyopathy (DCM)	05414	0.009	9	GABAergic synapse	04727	0.001
**10**	**Wnt signaling pathway**	**04310**	**0.009**	10	Regulation of actin cytoskeleton	04810	0.001
11	Fructose and mannose metabolism	00051	0.009	11	Aldosterone-regulated sodium reabsorption	04960	0.001
12	NOD-like receptor signaling pathway	04621	0.009	12	HTLV-I infection	05166	0.001
13	Autophagy – animal	04140	0.009	13	Prostate cancer	05215	0.001
14	Glycosphingolipid biosynthesis - lacto and neolacto series	00601	0.009	14	Systemic lupus erythematosus	05322	0.001
15	Arrhythmogenic right ventricular cardiomyopathy (ARVC)	05412	0.009	…			0.001
				**123**	**Wnt signalling pathway**	**04310**	**0.97**

'Wnt signaling pathway' and 'colorectal cancer' pathways shown in bold are expected to be impacted in GSE8671 dataset. These pathways are among the top-ranked pathways found by FoPA

According to the analysis accomplished by FoPA, six pathways (p-value < 0.05) are recognized as relevant to colorectal adenoma (GSE8671). The first one is the ‘*non-small cell lung cancer signaling pathway’*. This is consistent with a recent study [57] shown that the majority of genes for colon and lung cancer susceptibility are linked pair-wise and are likely identical or related.

The second identified one is the ‘*Intestinal immune network for IgA production’*. The differentially expressed genes of the present dataset show that the expression of the majority of genes in the ‘*intestinal immune network for IgA production’* pathway is lower than that in the normal mucosa. These include a series of human leukocyte antigen (HLA) class II genes (HLA-DOA, DPA1, DPB1, DQA1, DQA2, DQB1, DMB, DRA, DRB1, DRB3, DRB4, and DRB5). These genes encode major histocompatibility complex class II molecules in antigen presenting cells (B lymphocytes, dendritic cells, and macrophages), which are essential for the proliferation and differentiation of B cells [58]. Since IgA-secreting cells contribute to reducing inflammatory response which is a strong risk factor for the development of gastrointestinal adenocarcinomas, it is likely that the impairment of IgA production may drive further inflammatory responses and promote tumor growth. This is consistent with prior studies that showed the influence of IgA-secreting cells and B cells to colon tumors progression [59, 60].

The third identified pathway is ‘*Aldosterone-regulated sodium reabsorption’*. In this pathway, Aldosterone binds MR (Mineralocorticoid Receptor), which translocate into the nucleus and regulates gene transcription. A recent study [61] have demonstrated that decreased MR expression can contribute to angiogenesis and poor patient survival in colorectal malignancies and they show MR activation in the presence of a physiological amount of aldosterone exerts a negative role on angiogenesis.

The fourth pathway is ‘*Tight Junction’*(TJ) pathway. Claudin family proteins consisting of at least 24 members are essential for the formation of TJs and have a significant effect on the biological behavior of tumor progression. Previous studies have demonstrated the several claudin (claudin-1 [62, 63], claudin-3 [64, 65], claudin-4 [64], claudin-7 [65]) aberrant expression patterns in colorectal cancer. Among them, claudin-1 and claudin-2 overexpression are identified in the present dataset.

The fifth pathway is *‘p53 signaling pathway*’ where its dysfunction is highly prevalent in most cancers [66].

*Autism exome sequencing study*: Rubeis et al. [48] have analyzed exome sequencing of autism patients and healthy people and identified 22 autism-related genes. Here, these genes are considered as differentially expressed genes and the mutation rate of them as the probability of each gene instead of *t score* in differentially expressed genes analysis.

The results of applying FoPA to autism-related genes are shown in Table 5 (More results are given in Additional file 7). FoPA does not find any significant pathway (*p*-value < 0.05). However, five high ranked pathways worth to be considered as the likely related pathways to autism. Through reviews of literature, pieces of evidence are provided showing these pathways may be related to autism.

**Table 5:** results of applying FoPA to autism-related genes

	KEGG ID	KEGG pathway name	Score	P_FoPA_
1	05016	Huntington’s disease	2.1e-03	0.186
2	04742	Taste transduction	2e-04	0.22
3	05012	Parkinson’s disease	2.6e-06	0.23
4	05310	Asthma	1.7e-07	0.47
5	04727	GABAergic synapse	6.2e-05	0.48
6	04614	Renin-angiotensin system	3.01e-07	0.48

It has been reported that atypical processing of odor and taste stimuli is presented in autism spectrum disorders (ASD) [67, 68]. A study [69] examined the relationship between sensory responsiveness and social severity in children with high functioning ASD. Analyses revealed scores of oral sensory, olfactory, and touch as the strongest predictors of greater social impairment in autism.

Asthma is another identified KEGG pathway. Recently, researchers at Sydney University’s Brain and Mind Centre have published a study that shows a relationship between a mother’s active immune response during pregnancy to allergies and asthma and severe social impairment symptoms in children with autism [70].

GABAergic synapse may be one of the important relevant pathways to ASD. Several lines of evidence suggest that an impairment of GABAergic transmission contributes to the development of ASDs. GABAergic signaling dysfunction early in development leads to a severe excitatory/inhibitory unbalance in neuronal circuits, a condition that may account for some of the behavioral deficits observed in ASD patients [71].

The last pathway in the list is the Renin-angiotensin system (RAS). This pathway has been hypothesized to have a pivotal role in some neurodegenerative diseases, such as Parkinson, Alzheimer, Huntington and Multiple Sclerosis (MS) [72].

Angiotensin-converting enzyme (ACE) is the essential enzyme in this pathway which plays a major role in the degeneration of a family of neurotransmitters in the central nervous system (CNS). The implication of neurotransmitters in psychiatric disorders is supported by their function in the regulation of emotions, cognition, behavior, and memory which are disrupted in autism [73].

This analysis highlights the role of *Renin-angiotensin* system and *GABAergic synapse* pathways in ASD. It can be concluded that there may exist relations between *GABAergic synapse* pathway and *RAS* in the development of autism. Further genetic studies can support this finding.

### Systematic analysis of the possible bias

**Fig. 9:**
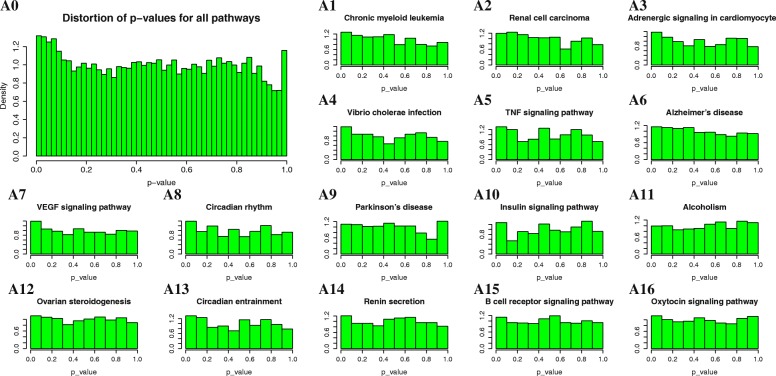
Null distributions of p-values produced by FoPA: The large panel on the left (A0), displays the distribution of p-values cumulated from all KEGG signaling pathways. The smaller panels on the right (A1-A16) display the p-values distributions of individual pathways, which are extreme cases sorted based on the distribution’s mean

To analysis, the possible bias of the FoPA, a total of 1000 resampled datasets from 41 control samples of 4 Alzheimer’s datasets (GSE5281_EC, GSE5281_HIP, GSE5281_VCX, and GSE16759) is generated. Some of them (18) are randomly labeled as disease and the remaining marked as normal samples. This procedure is repeated 500 times to create different groups of 18 disease and 17 control samples. To eliminate the effect of the group size 100 datasets consisting of 10 control and 10 diseases, 200 datasets consisting of 10 control and 20 diseases and 200 datasets consisting of 20 control and 10 diseases samples are also generated. The *p*-values of KEGG signaling pathways for each of the datasets are calculated using FoPA. The results, Fig. 9, indicate that the distributions of p-values cumulated from all KEGG signaling pathways are a bit biased toward zero. However, it’s not so much that it can affect the performance of the FoPA. The top 16 most biased pathways sorted by their distributions means have also shown in Fig. 9. It is showed that the distributions of p-values produced by FoPA are reasonably uniform for each of these pathways, while the results of the analysis in [49] have shown that PADOG and SPIA are biased towards generating lower p-values for some of the signaling pathways.

### The impact of the pathway’s incompleteness and noise on FoPA’s performance

**Fig. 10:**
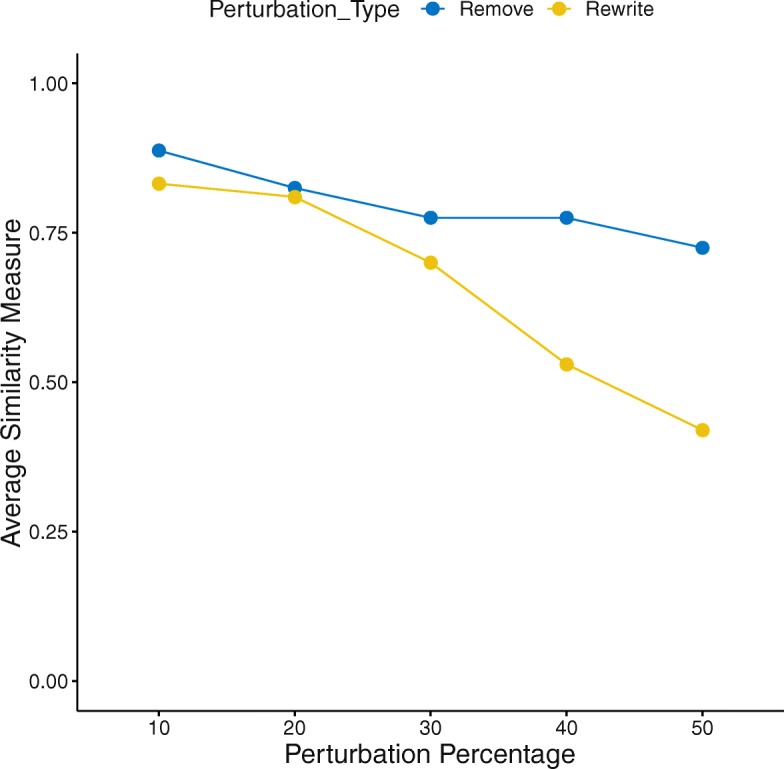
The impact of perturbation on the performance of FoPA*:* each time a portion of interaction in a pathway are randomly removed or rewired, FoPA is re-applied to infer is the generated pathway is identified the same as the main pathway (both relevant or both not relevant). The similarity measure is the number of the generated pathways identified the same as the main pathway

Pathway database incompleteness and noise are mimicked by randomly removing or rewiring a portion (e.g., 10, 20, 30, 40, and 50%) of the interactions in a pathway. At each portion of edge removal or rewriting for each pathway, the experiment is repeated 100 times. The similarity measure for each pathway is computed as the number of the generated pathways identified the same as the main pathway (both relevant or both not relevant) to the condition under study. The average of these measures for each pathway at each portion is illustrated in Fig. 10. The results have shown that the average similarity drop with an increase of perturbations. Interaction rewriting has a more significant effect on FoPA than removal does.

## Conclusion

In this study, a new pathway analyzing approach is introduced, that uses formal methods to rank pathways according to their relevance to a given clinical condition (e.g., disease). To the best of the authors’ knowledge, it is the first attempt in using the formal methods in solving such a problem. Formal modeling has many advantages over the modeling by graphs. It helps researchers to express any relations among biological components involved in an interaction. This helps to create a reliable model of signaling pathways that can be effective in reducing the false results in pathway analysis studies. The proposed tool named FoPA is constructed based on this new approach. We have compared FoPA with three other analysis methods, two topology-based (CePa, SPIA) and one gene set-based (PADOG). These methods are chosen regarding their performance in previous comparisons.

Some techniques are used to evaluate the FoPA’s performance and compare it with other methods. We have assessed the results considering both rankings and *P*-values of the target pathways. The results indicate that PADOG and FoPA are able to prioritize target pathways with high sensitivity when compared with CePa and SPIA. However, considering the target pathway technique performance is not enough. Thus, the simulated false inputs (permuted class labels and decoy pathways) are created as a set of negative controls to measure the false-positive rate of the methods. The number of significant pathways identified by giving permuted class labels to FoPA is less than the other three methods; that is, FoPA differentiates significantly between actual and random clinical data. Moreover, the area under the curve ROC is greater in FoPA compared with PADOG and CePa, which indicates that FoPA excludes more decoy pathways from real ones.

The results of the methods on independent colorectal cancer datasets indicate that, FoPA identifies more overlaps than PADOG and SPIA at almost all rank thresholds and its finding is in line with other researches. Although CePa has found more overlaps in some rank thresholds, these overlaps appear to have false pathways. The consistency analysis is also performed on a group of dependent datasets (datasets made by resampling). The average overlapped pathways found by FoPA on these datasets is more than that of other methods.

We also have demonstrated that there is no systematic bias in FoPA that makes some pathways detected as significant regardless of the input differentially expressed genes.

As an application of FoPA, we apply it to a list of autism-related genes and show that FoPA can discover pathways relevant to autism. This analysis highlights the role of *Renin-angiotensin* system and *GABAergic synapse* pathways in ASD.

These lines of evidence well demonstrate FoPA’s advantage over the other methods. One of the disadvantages of FoPA may be its high running time compared with available statistical methods. Though this running time is tolerable, it will be decreased through the improvement made in our modeling and also in formal verification techniques.

The other disadvantage of FoPA is that it treats pathways as independent entities, and gives more focus on pathway-specific genes rather than overlapped genes among pathways. While some of these overlapped genes will lead to “crosstalk” phenomenon that could influence other pathways. As a result, considering cross-talk genes and inter-pathway relations may lead to better performance of FoPA.

It is worth mentioning that FoPA is the first attempt of using formal methods in pathway analysis. So, by adding other details in model specification and considering some other aspects such as pathway’s cross-talk, the result of the experiments would be improved significantly.

## Additional files

Additional file1: The file contains the additional details on the following: i) formal definition of Markov chains ii) probability measure of Markov chains iii) reachability probabilities iv) a toy example showing how the model checking based approach works. IV) Results of FoPA on different disease datasets. (DOCX 3053 kb)

Additional file 2: The 36 datasets used as a benchmark in the target pathway technique. (XLSX 11 kb)

Additional file 3: The results of the target pathway technique (ranks and *P*-values of the target pathways associated with each benchmark dataset) for each of four methods (FoPA, PADOG, CePa, and SPIA). (XLSX 15 kb)

Additional file 4: The results of four methods (FoPA, PADOG, CePa, and SPIA) on four colorectal cancer datasets (GSE4107, GSE9348, GSE4183, GSE23878). (XLSX 120 kb)

Additional file 5: The results of four methods (FoPA, PADOG, CePa, and SPIA) on GSE8671 dataset. (XLSX 37 kb)

Additional file 6: List of the differentially expressed genes identified for GSE8671 dataset. (XLSX 1282 kb)

Additional file 7: The results of FoPA on autism genes. (XLSX 416 kb)

## Abbreviations

AUC: Area under curve
CePa: Centrality based pathway analysis
CTMC: Continuous-time Markov chain
DEG: Differentially expressed gene
DTMC: Discrete-time Markov chains
FoPA: Formal model based checking pathway analysis
MDP: Markov decision processes
PADOG: Pathway analysis with down-weighting overlapping genes
SPIA: Signaling pathway impact analysis
