# Supplementary material for: FoPA: identifying perturbed signaling pathways in clinical conditions using formal methods
Source: BMC Bioinformatics. 2019 Feb 26;20:92. doi: 10.1186/s12859-019-2635-6 (PMC6390332; doi:10.1186/s12859-019-2635-6)
Supplement: Supplementary file 1 — The file contains the additional details on the following: i) formal definition of Markov chains ii) probability measure of Markov chains iii) reachability probabilities iv) a toy example showing how the model checking based approach works. IV) Results of FoPA on different disease datasets. (DOCX 3053 kb) [file 12859_2019_2635_MOESM1_ESM.docx]

Supplementary Material for: FoPA: Identifying perturbed signaling pathways in clinical conditions using formal methods

Fatemeh Mansoori^1^, Maseud Rahgozar^1*^, Kaveh Kavousi^2*^

^1^Database Research Group, Control and Intelligent Processing Center of Excellence, School of Electrical and Computer Engineering, University of Tehran, Tehran, Iran

^2^Complex Biological Systems and Bioinformatics Lab, Institute of Biochemistry and Biophysics (I.B.B), University of Tehran, Tehran, Iran

*Email: [kkavousi@ut.ac.ir](mailto:kkavousi@ut.ac.ir)

**1-1 Discrete-Time Markov Chain (MC)**

A (discrete-time) Markov chain is a tuple $\mathcal{M=(}S, P, I, AP, L)$ where

- S is a countable, nonempty set of states,
- $P:S*S \underset{\to}{} \left[ 0,1 \right]$ is the transition probability function such that for all states s :

$$\sum_{s^{'}\in S} P(s,s^{'})=1$$

- $I:S \underset{\to}{}\left[ 0,1 \right] is the initial distribution, such that \sum_{s\in S} I(s)=1$
- AP is a set of atomic propositions and $L :S\underset{\to}{}2^{AP}$ a labeling function.

The transition probability function *P* specifies for each state *s* the probability *P(s,s')* of moving from *s* to *s'* in one step, i.e., by a single transition. The constraint imposed on *P* ensures that *P* is a distribution.

**1-2 Probability Measure of Markov chain**

Let Paths($\mathcal{M}$) denotes the set of all infinite sequences $s_{0}s_{1}s_{2}\ldots\in S^{\omega}$ such that P(s_i_,s_i+1_) > 0 for all i≥0. and Pathsfin($\mathcal{M}$) denotes the set of finite path fragments s_0_s_1_…s_n_ where n ≥ 0 and P(s_i_,s_i+1_) > 0 for 0 ≤ i < n. The cylinder set of $\hat{\pi}=s_{0}\ldots s_{n}\in{Paths}_{fin}\mathcal{(M)}$ is defined as :

$$Cyl\left( \hat{\pi} \right)=\{\pi\epsilon Paths\mathcal{(M)|}\hat{\pi}\in prefix(\pi)$$

The cylinder set spanned by the finite path $\hat{\pi}$ thus consists of all infinite paths that start with $\hat{\pi}.$

In order to associate probabilities to events in Markov chains, a probability space is associated with $\mathcal{M}$ where the infinite paths of $\mathcal{M}$ play the role of outcomes.

It is proved in [1] that there exists a unique probability measure Pr with $\mathcal{M}$ where the probabilities for the cylinder sets (i.e. the events) are given by

$$\Pr\left( Cyl\left( s_{0}\ldots s_{n} \right) \right)=P\left( s_{0}\ldots s_{n} \right)=\prod_{0\leq i<n} P\left( s_{i},s_{i+1} \right).$$

**1-3 Reachability Probabilities**

LTL-like notations are used to describe events in Markov chains. Let ◊B, the event of interest denotes the event to reach some states in B eventually. Thus, the set of all paths satisfied ◊B is given by ${Path}_{fin}\mathcal{(M)\cap}\left( S\backslash B \right)^{*}B$. The cylinder sets of these paths are pairwise disjoint, thus the probability of eventually reaching B is giving by

$$\Pr\left( ◊B \right)= \sum_{s_{0}\ldots s_{n}\in{Paths}_{fin}(M)\cap{(S\backslash B)}^{*}B} \Pr\left( Cyl\left( s_{0}\ldots s_{n} \right) \right)$$

$$= \sum_{s_{0}\ldots s_{n}\in{Paths}_{fin}(M)\cap{(S\backslash B)}^{*}B} P(s_{0}\ldots s_{n})$$

**2 A toy example**

A toy example of a small fragment of a pathway is shown in Figure 1 wherein *P_1_,…,P_4_* indicate the probability of interactions. For modeling this pathway, to each protein, a variable is dedicated indicating its state. Suppose three states *a*, *a'*, and *n* is dedicated to each variable where *a* indicates the protein is activated, *a'* indicates it is not activated and *n* means the protein-coding gene is not expressed. The values of all variables indicate the state of the system. Rules are defined by PRISM languages, to specify how the states of the system change over time. These rules are tabulated in Table 1.


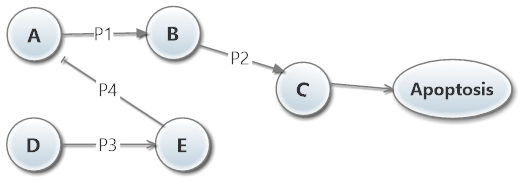


Figure 1- A toy example of a small fragment of a pathway

Table 1- PRISM rules for toy example

| No. |  |  | PRISM Language Specification |
| --- | --- | --- | --- |
| (1) | A→B | Activation | $\left[ \right] A=a \& B!=n \to{prob}_{active}:(B'=a)+(1-{prob}_{active}):(B'=n);$ |
| (2) | A $⊣$ B | Inhibition | $\left[ \right] A=a \& B=a \underset{\to}{} {prob}_{inhibit}:(B'=a')+(1-{prob}_{inhibit2}):(B'=n);$ |

Suppose that all the protein-coding genes *A*, *B*, *C*, *D,* and *E* are expressed and proteins *A* and *D* are activated, thus the states of the system and how it evolves from one state to another is shown in Figure 2 wherein the initial state is shown by a thicker border.

Figure 2-States of the system for the toy example

Suppose that our interest is to compute the probability of reaching the *apoptosis*. And also suppose that active protein C result in *apoptosis*. Thus the desired property of the model is defined by the LTL property ◊(C=a). The desired states are shown in red in Figure 2. For this event, the finite path fragment s_0_…s_n_ with s_i_ **|≠** (C=a) for 0 ≤ *i* < *n* and s_n_ **|=** (C=a) are of interest. These path fragments are as follows:

- **π_1_ = s_0_s_1_s_20_**
- **π_2_ = s_0_s_1_s_9_s_19_**
- **π_3_ = s_0_s_1_s_9_s_21_s_16_**
- **π_4_ = s_0_s_2_s_9_s_19_**
- **π_5_ = s_0_s_2_s_9_s_22_s_25_^ω^**
- **π_6_ = s_0_s_3_s_12_s_13_^ω^**

The probabilities of the cylinder sets spanned by π_1_,…, π_6_ are:

- $\Pr\left( Cyl\left( \pi_{1} \right) \right)=\frac{P_{1}}{2}.\frac{P_{2}}{2}$
- $\Pr\left( Cyl\left( \pi_{2} \right) \right)=\frac{P_{1}}{2}.\frac{P_{3}}{2}.\frac{P_{2}}{2}$
- $\Pr\left( Cyl\left( \pi_{3} \right) \right)=\frac{P_{1}}{2}.\frac{P_{3}}{2}.\frac{P_{4}}{2}P_{2}$
- $\Pr\left( Cyl\left( \pi_{4} \right) \right)=\frac{P_{3}}{2}.\frac{P_{1}}{2}.\frac{P_{2}}{2}$
- $\Pr\left( Cyl\left( \pi_{5} \right) \right)=\frac{P_{3}}{2}.\frac{P_{1}}{2}.\frac{{1-P}_{4}}{2}P_{2}$
- $\Pr\left( Cyl\left( \pi_{6} \right) \right)=\frac{{1-P}_{3}}{2}.P_{1}P_{2}$

Hence,

$$\Pr\left( ◊(C=a) \right)=\frac{P_{1}}{2}.\frac{P_{2}}{2}+\frac{P_{1}}{2}.\frac{P_{3}}{2}.\frac{P_{2}}{2}+\frac{P_{1}}{2}.\frac{P_{3}}{2}.\frac{P_{4}}{2}.P_{2}+\frac{P_{3}}{2}.\frac{P_{1}}{2}.\frac{P_{2}}{2}+\frac{P_{3}}{2}.\frac{P_{1}}{2}.\frac{{1-P}_{4}}{2}P_{2}+\frac{{1-P}_{3}}{2}.P_{1}P_{2}$$

$$=\frac{P_{1}}{2}.\frac{P_{2}}{2}+\frac{P_{1}}{2}.\frac{P_{2}}{2}.P_{3}+\frac{P_{1}}{2}.\frac{P_{3}}{2}.\frac{P_{4}}{2}.P_{2}+\frac{P_{3}}{2}.\frac{P_{1}}{2}.\frac{{1-P}_{4}}{2}P_{2}+\frac{{1-P}_{3}}{2}.P_{1}P_{2}$$

$$=\frac{P_{1}}{2}.\frac{P_{2}}{2}(1+P_{3}+.\frac{{P_{3}P}_{4}}{2}+\frac{{P_{3}-P_{3}P}_{4}}{2}+{2-2P}_{3})$$

$$=\frac{P_{1}}{2}.\frac{P_{2}}{2}(3-\frac{P_{3}}{2})$$

$$=\frac{{3P}_{1}P_{2}}{4}-\frac{P_{1}P_{2}P_{3}}{8}$$

**4 Tables 1-5**

**5 References**

1. Baier, C., Katoen, J. P., Larsen, K. G. Principles of model checking-MIT press (2008).

____________________________________________________________________________________________________

Table 1. FoPA results on the GSE21354 (gene expression profiling of three type of grade II gliomas) dataset.

| Rank | Pathway name | Pathway ID | Pathway Score | P_FoPA_ |
| --- | --- | --- | --- | --- |
| 1 | Chemokine signaling pathway | 04062 | 0.069526 | 0 |
| 2 | PI3K-Akt signaling pathway | 04151 | 0.068655 | 0 |
| 3 | Vibrio cholerae infection | 05110 | 0.062147 | 0 |
| 4 | Sphingolipid signaling pathway | 04071 | 0.030627 | 0 |
| 5 | Colorectal cancer | 05210 | 0.029319 | 0 |
| 6 | Adherens junction | 04520 | 0.026779 | 0 |
| 7 | Renin secretion | 04924 | 0.001138 | 0 |
| 8 | Endometrial cancer | 05213 | 0.00052 | 0 |
| 9 | Glioma | 05214 | 0.000315 | 0.007752 |
| 10 | MAPK signaling pathway | 04010 | 0.039615 | 0.009009 |
| 11 | Ovarian steroidogenesis | 04913 | 0.046914 | 0.009709 |
| 12 | GnRH signaling pathway | 04912 | 0.034805 | 0.009709 |
| 13 | Amphetamine addiction | 05031 | 0.034663 | 0.009709 |
| 14 | Regulation of lipolysis in adipocytes | 04923 | 0.013704 | 0.009709 |
| 15 | Olfactory transduction | 04740 | 0.007682 | 0.009709 |
| 16 | Cytosolic DNA-sensing pathway | 04623 | 0.002501 | 0.009709 |
| 17 | Circadian entrainment | 04713 | 0.000628 | 0.009709 |
| 18 | Dilated cardiomyopathy | 05414 | 0.040888 | 0.013699 |
| 19 | Prostate cancer | 05215 | 0.012245 | 0.015152 |
| 20 | Renal cell carcinoma | 05211 | 0.040676 | 0.016598 |
| 21 | cAMP signaling pathway | 04024 | 0.107354 | 0.019417 |
| 22 | Adrenergic signaling in cardiomyocytes | 04261 | 0.098272 | 0.019417 |
| 23 | Insulin secretion | 04911 | 0.02958 | 0.019417 |
| 24 | Fc gamma R-mediated phagocytosis | 04666 | 0.028572 | 0.019417 |
| 25 | NF-kappa B signaling pathway | 04064 | 0.01267 | 0.019417 |

Table 2. FoPA results on the GSE18842 (Gene expression analysis of human lung cancer (NSCLC)) dataset.

| Rank | Pathway name | Pathway ID | Pathway Score | P_FoPA_ |
| --- | --- | --- | --- | --- |
| 1 | Serotonergic synapse | 04726 | 0.051053 | 0 |
| 2 | Adherens junction | 04520 | 0.02632 | 0 |
| 3 | Thyroid cancer | 05216 | 0.013175 | 0 |
| 4 | Acute myeloid leukemia | 05221 | 0.01734 | 0.005556 |
| 5 | Non-small cell lung cancer | 05223 | 0.001294 | 0.006494 |
| 6 | Leukocyte transendothelial migration | 04670 | 0.057689 | 0.009709 |
| 7 | Salmonella infection | 05132 | 0.047719 | 0.019417 |
| 8 | Sphingolipid signaling pathway | 04071 | 0.018572 | 0.019417 |
| 9 | Arrhythmogenic right ventricular cardiomyopathy (ARVC) | 05412 | 0.015896 | 0.019417 |
| 10 | NF-kappa B signaling pathway | 04064 | 0.014789 | 0.019417 |
| 11 | Vasopressin-regulated water reabsorption | 04962 | 0.009523 | 0.019417 |
| 12 | Alzheimer's disease | 05010 | 0.015697 | 0.025 |
| 13 | Tight junction | 04530 | 0.016661 | 0.029126 |
| 14 | cGMP-PKG signaling pathway | 04022 | 0.064092 | 0.038462 |
| 15 | Prolactin signaling pathway | 04917 | 0.025574 | 0.038835 |
| 16 | Staphylococcus aureus infection | 05150 | 0.014992 | 0.038835 |
| 17 | TGF-beta signaling pathway | 04350 | 0.002029 | 0.038835 |
| 18 | Melanoma | 05218 | 0.000122 | 0.038835 |
| 19 | PPAR signaling pathway | 03320 | 0.20493 | 0.044643 |
| 20 | Legionellosis | 05134 | 0.044126 | 0.048544 |
| 21 | Pertussis | 05133 | 0.018143 | 0.048544 |
| 22 | Melanogenesis | 04916 | 0.007039 | 0.048544 |
| 23 | Chronic myeloid leukemia | 05220 | 0.015419 | 0.05 |
| 24 | VEGF signaling pathway | 04370 | 0.008828 | 0.058252 |
| 25 | Pancreatic secretion | 04972 | 4.69E-05 | 0.058252 |

Table 3. FoPA results on the GSE14924-CD8 (gene expression changes in T cells from patients presenting with Acute myeloid leukemia (AML)) dataset.

| Rank | Pathway name | Pathway ID | Pathway Score | P_FoPA_ |
| --- | --- | --- | --- | --- |
| 1 | Cocaine addiction | 05030 | 0.104147 | 0 |
| 2 | Focal adhesion | 04510 | 0.098227 | 0 |
| 3 | Apoptosis | 04210 | 0.049035 | 0 |
| 4 | Adherens junction | 04520 | 0.04903 | 0 |
| 5 | Sphingolipid signaling pathway | 04071 | 0.038639 | 0 |
| 6 | Colorectal cancer | 05210 | 0.035645 | 0 |
| 7 | PI3K-Akt signaling pathway | 04151 | 0.033333 | 0 |
| 8 | Non-small cell lung cancer | 05223 | 0.001071 | 0.006494 |
| 9 | Toll-like receptor signaling pathway | 04620 | 0.147499 | 0.009709 |
| 10 | Insulin signaling pathway | 04910 | 0.07035 | 0.009709 |
| 11 | Renin secretion | 04924 | 0.000556 | 0.009709 |
| 12 | Carbohydrate digestion and absorption | 04973 | 0.000276 | 0.009709 |
| 13 | Acute myeloid leukemia | 05221 | 0.012733 | 0.011111 |
| 14 | Chronic myeloid leukemia | 05220 | 0.024906 | 0.016667 |
| 15 | Epithelial cell signaling in Helicobacter pylori infection | 05120 | 0.02847 | 0.019417 |
| 16 | TNF signaling pathway | 04668 | 0.021361 | 0.019417 |
| 17 | cAMP signaling pathway | 04024 | 0.066404 | 0.029126 |
| 18 | B cell receptor signaling pathway | 04662 | 0.042301 | 0.029126 |
| 19 | Ovarian steroidogenesis | 04913 | 0.037724 | 0.029126 |
| 20 | Fc gamma R-mediated phagocytosis | 04666 | 0.025344 | 0.029126 |
| 21 | Insulin secretion | 04911 | 0.019673 | 0.029126 |
| 22 | Olfactory transduction | 04740 | 0.003878 | 0.029126 |
| 23 | Alzheimer's disease | 05010 | 0.013954 | 0.03 |
| 24 | Glioma | 05214 | 0.000268 | 0.031008 |
| 25 | Adrenergic signaling in cardiomyocytes | 04261 | 0.053274 | 0.038835 |

Table 4. FoPA results on the GSE7305 (Gene expression analysis of human endometriosis) dataset.

| Rank | Pathway name | Pathway ID | Pathway Score | P_FoPA_ |
| --- | --- | --- | --- | --- |
| 1 | p53 signaling pathway | 04115 | 0.046592 | 0.087379 |
| 2 | Bile secretion | 04976 | 0.004745 | 0.087379 |
| 3 | NF-kappa B signaling pathway | 04064 | 0.000963 | 0.087379 |
| 4 | Taste transduction | 04742 | 0.000284 | 0.15534 |
| 5 | Neurotrophin signaling pathway | 04722 | 0.00671 | 0.165049 |
| 6 | Vasopressin-regulated water reabsorption | 04962 | 0.000448 | 0.184466 |
| 7 | Intestinal immune network for IgA production | 04672 | 0.003643 | 0.194175 |
| 8 | Legionellosis | 05134 | 0.005188 | 0.203883 |
| 9 | Regulation of lipolysis in adipocytes | 04923 | 0.001038 | 0.203883 |
| 10 | Chronic myeloid leukemia | 05220 | 0.001939 | 0.205556 |
| 11 | Staphylococcus aureus infection | 05150 | 0.003138 | 0.213592 |
| 12 | Complement and coagulation cascades | 04610 | 0.00406 | 0.223301 |
| 13 | Endometrial cancer | 05213 | 4.37E-05 | 0.251908 |
| 14 | Mineral absorption | 04978 | 0.000945 | 0.252427 |
| 15 | Adherens junction | 04520 | 0.005026 | 0.259259 |
| 16 | Colorectal cancer | 05210 | 0.002854 | 0.260204 |
| 17 | Leukocyte transendothelial migration | 04670 | 0.013213 | 0.262136 |
| 18 | Thyroid cancer | 05216 | 0.001404 | 0.271845 |
| 19 | Tight junction | 04530 | 0.001056 | 0.271845 |
| 20 | Hedgehog signaling pathway | 04340 | 0.000435 | 0.271845 |
| 21 | Systemic lupus erythematosus | 05322 | 0.000669 | 0.291262 |
| 22 | Viral myocarditis | 05416 | 0.002062 | 0.3 |
| 23 | Huntington's disease | 05016 | 0.000645 | 0.3 |
| 24 | Renin-angiotensin system | 04614 | 3.58E-05 | 0.31068 |
| 25 | Autoimmune thyroid disease | 05320 | 0.001191 | 0.320388 |

Table 5. FoPA results for GSE5281-HIP (Gene expression analysis of the Alzaymer's disease) dataset.

| Rank | Pathway name | Pathway ID | Pathway Score | P_FoPA_ |
| --- | --- | --- | --- | --- |
| 1 | Regulation of actin cytoskeleton | 04810 | 0.0477 | 0 |
| 2 | Pancreatic secretion | 04972 | 0.000151 | 0.029126 |
| 3 | Thyroid hormone signaling pathway | 04919 | 0.114046 | 0.038835 |
| 4 | Tight junction | 04530 | 0.011329 | 0.038835 |
| 5 | PI3K-Akt signaling pathway | 04151 | 0.025527 | 0.04 |
| 6 | Vasopressin-regulated water reabsorption | 04962 | 0.00404 | 0.048544 |
| 7 | Alzheimer's disease | 05010 | 0.009694 | 0.05 |
| 8 | Cocaine addiction | 05030 | 0.030957 | 0.058252 |
| 9 | NF-kappa B signaling pathway | 04064 | 0.002779 | 0.058252 |
| 10 | Colorectal cancer | 05210 | 0.010499 | 0.061224 |
| 11 | Parkinson's disease | 05012 | 4.6E-05 | 0.074074 |
| 12 | Prostate cancer | 05215 | 0.005005 | 0.075758 |
| 13 | Epithelial cell signaling in Helicobacter pylori infection | 05120 | 0.010533 | 0.07767 |
| 14 | Amyotrophic lateral sclerosis (ALS) | 05014 | 0.005676 | 0.087379 |
| 15 | Sphingolipid signaling pathway | 04071 | 0.009817 | 0.097087 |
| 16 | Olfactory transduction | 04740 | 0.00245 | 0.097087 |
| 17 | Retrograde endocannabinoid signaling | 04723 | 6.6E-06 | 0.097087 |
| 18 | Adrenergic signaling in cardiomyocytes | 04261 | 0.028054 | 0.106796 |
| 19 | Vibrio cholerae infection | 05110 | 0.023295 | 0.106796 |
| 20 | Serotonergic synapse | 04726 | 0.021521 | 0.106796 |
| 21 | Non-alcoholic fatty liver disease (NAFLD) | 04932 | 0.005378 | 0.106796 |
| 22 | Regulation of lipolysis in adipocytes | 04923 | 0.004007 | 0.106796 |
| 23 | Adherens junction | 04520 | 0.015904 | 0.111111 |
| 24 | Ovarian steroidogenesis | 04913 | 0.024597 | 0.116505 |
| 25 | Small cell lung cancer | 05222 | 0.015301 | 0.116505 |
